# Tympanoplasty – news and new perspectives

**DOI:** 10.3205/cto000146

**Published:** 2017-12-18

**Authors:** Marcus Neudert, Thomas Zahnert

**Affiliations:** 1 Technical University of Dresden, Germany; 2 Medical Faculty “Carl Gustav Carus”, Dresden, Germany; 3 Department of Otolaryngology, Head and Neck Surgery, University Hospital of Dresden, Germany

**Keywords:** tympanoplasty, ossiculoplasty, quality control, outcome parameters, quality of life, middle ear prosthesis, PORP, TORP, middle ear mechanics

## Abstract

Techniques and biomaterials for reconstructive middle ear surgery are continuously and steadily developing. At the same time, clinical post-surgery results are evaluated to determine success or failure of the therapy. Routine quality assessment and assurance is of growing importance in the medical field, and therefore also in middle ear surgery. The exact definition and acquisition of outcome parameters is essential for both a comprehensive and detailed quality assurance. These parameters are not the audiological results alone, but also additional individual parameters, which influence the postoperative outcome after tympanoplasty. Selection of patients and the preoperative clinical situation, the extent of the ossicular chain destruction, the chosen reconstruction technique and material, the audiometric frequency selection and the observational interval are only some of them. If these parameters are not well documented, the value of comparative analyses between different studies is very limited. The present overview aims at describing, comparing, and evaluating some of the existing assessment and scoring systems for middle ear surgery. Additionally, new methods for an intraoperative quality assessment in ossiculoplasty and the postoperative evaluation of suboptimal hearing results with imaging techniques are available. In the area of implant development, functional elements were integrated in prostheses to enable not only good sound transmission but also compensation of occurring atmospheric pressure changes. In combination with other components for ossicular repair, they can be used in a modular manner, which so far show experimentally and clinically promising results.

## 1 Introduction

Since the first reports about the reconstruction of the sound conduction apparatus by Wullstein and Zöllner [1], [2], the measurement techniques and the research in biomaterials continued developing and fundamental knowledge was included in reconstructive middle ear surgery. The review articles of the last years extensively discussed the topics of biomechanics of the middle ear [3], [4], [5], [6], of prostheses and prosthetic materials [7], [8], [9], and of surgery as well as reconstruction techniques [4], [6], [7], [10], [11]. The focus of the present article will be placed on the influence factors and the assessment of outcome parameters after tympanoplasty. The desire to achieve more evidence is not only reflected in the increasing number of available guidelines, but it also requires well-conceived and detailed studies. For reconstructive middle ear surgery, this means that the outcome of tympanoplasty cannot only be measured based on the reduction of conductive hearing loss. Moreover, the most important parameters that evidently influence the postoperative outcome, must be identified and acknowledged. 

In a first step, biological and disease-specific influence factors will be discussed that have an impact on the postoperative hearing result. The consideration of those parameters plays an important role in the description and stratification of the study population because only in that way, comparison between the different studies is possible and applicable. Afterwards, the evaluation parameters that may determine the success of middle ear surgeries, will be in the focus. Only by selecting and describing the evaluation parameters in detail, the results may be analyzed and interpreted regarding their significance. The lack of acknowledged national and international standards makes it difficult to compare different studies so that meta-analyses cannot be performed effectively. In addition, new developments of intra- and postoperative quality assessment and assurance will be presented and evaluated. Finally, some new developments of middle ear prostheses and new achievements in ossiculoplasty will be dealt with.

## 2 Quality assessment in reconstructive middle ear surgery

**Figure 1:**
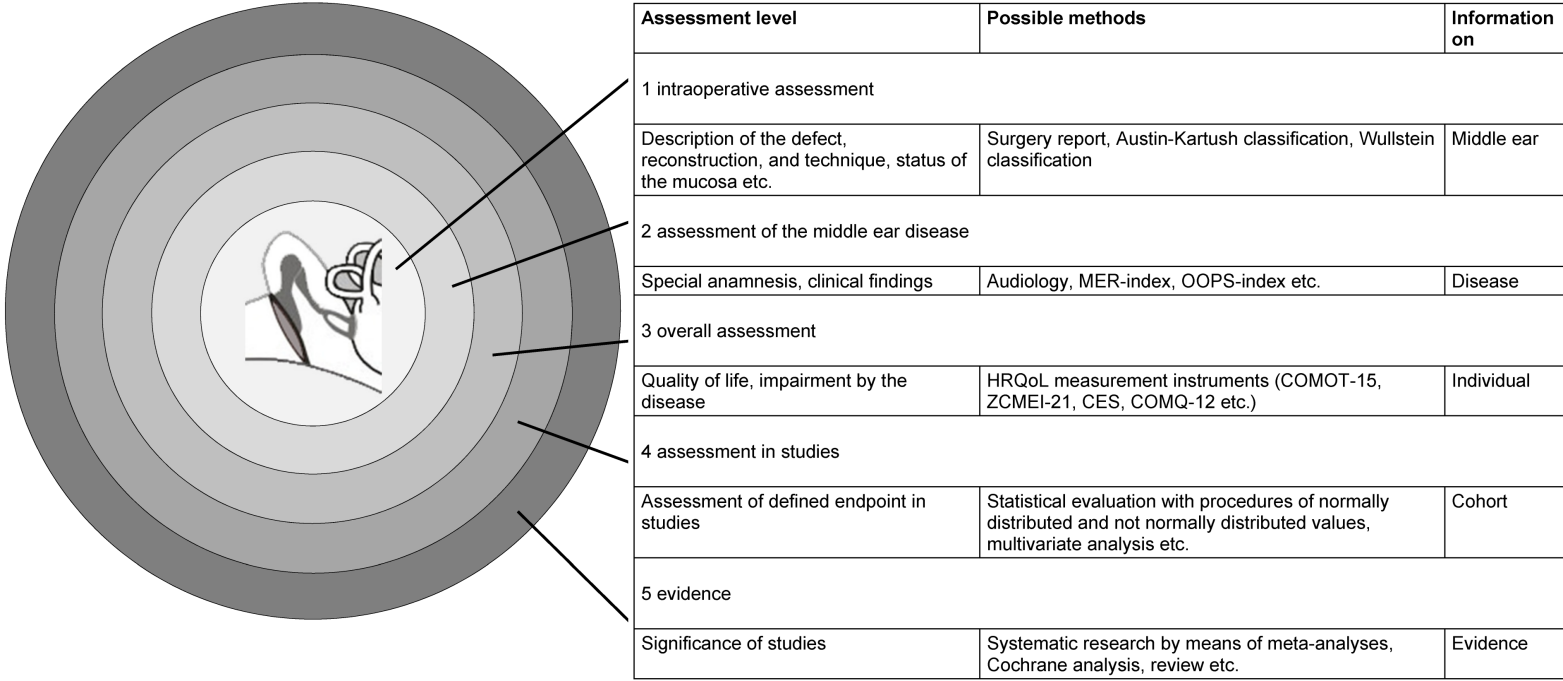
Scheme of the assessment levels of tympanoplasty or ear surgery. By triangulation of assessment instruments (possible methods), a more exact statement can be made with an increasing number of methods. As of level 4, the results of single patients are considered in the context of studies. The more detailed the characterization was performed on the previous levels, the more exact and valid are the conclusions. (OP: surgery; MER: middle ear risk; OOPS: ossiculoplasty outcome parameter staging; HRQoL: health-related quality of life; COMOT-15: chronic otitis media outcome test 15; ZCMEI-21: Zurich chronic middle ear inventory; CES: chronic ear survey; COMQ-12: chronic otitis media questionnaire 12).

In the last years, increasing efforts regarding quality assessment and assurance have been integrated in reconstructive middle ear surgery. Besides the clinical and audiological outcome parameters after tympanoplasty also patient-related aspects such as the health-related quality of life (HRQoL) play an increasing role. In order to describe the quality in a comprehensive and differentiated way, its influence factors and their documentation must be transparently determined. The assessment of success or failure of tympanoplasty for every individual patient seems to be manageable, well understandable, and communicable. But already when considering the individual patient, the intraoperative site or the postoperative hearing result do not allow a complete statement about the outcome. The more existing assessment methods are applied, the more extensive and valid is the possible statement or an extrapolation to future results and the comparability with other series. Figure 1 (Fig. 1) presents a model of assessment levels of middle ear surgery. Hereby, a triangulation of measurement instruments leads to a status description that becomes more extensive with every step until the individual patient is included in a study population at level 4. If the quality criteria of this population are sufficient, they may provide even more statements as part of meta-analyses. In this context, generalizations and conclusions that are made based on gradations without methodical substantiation, are problematic, however, unfortunately they are often found.

**Figure 2:**
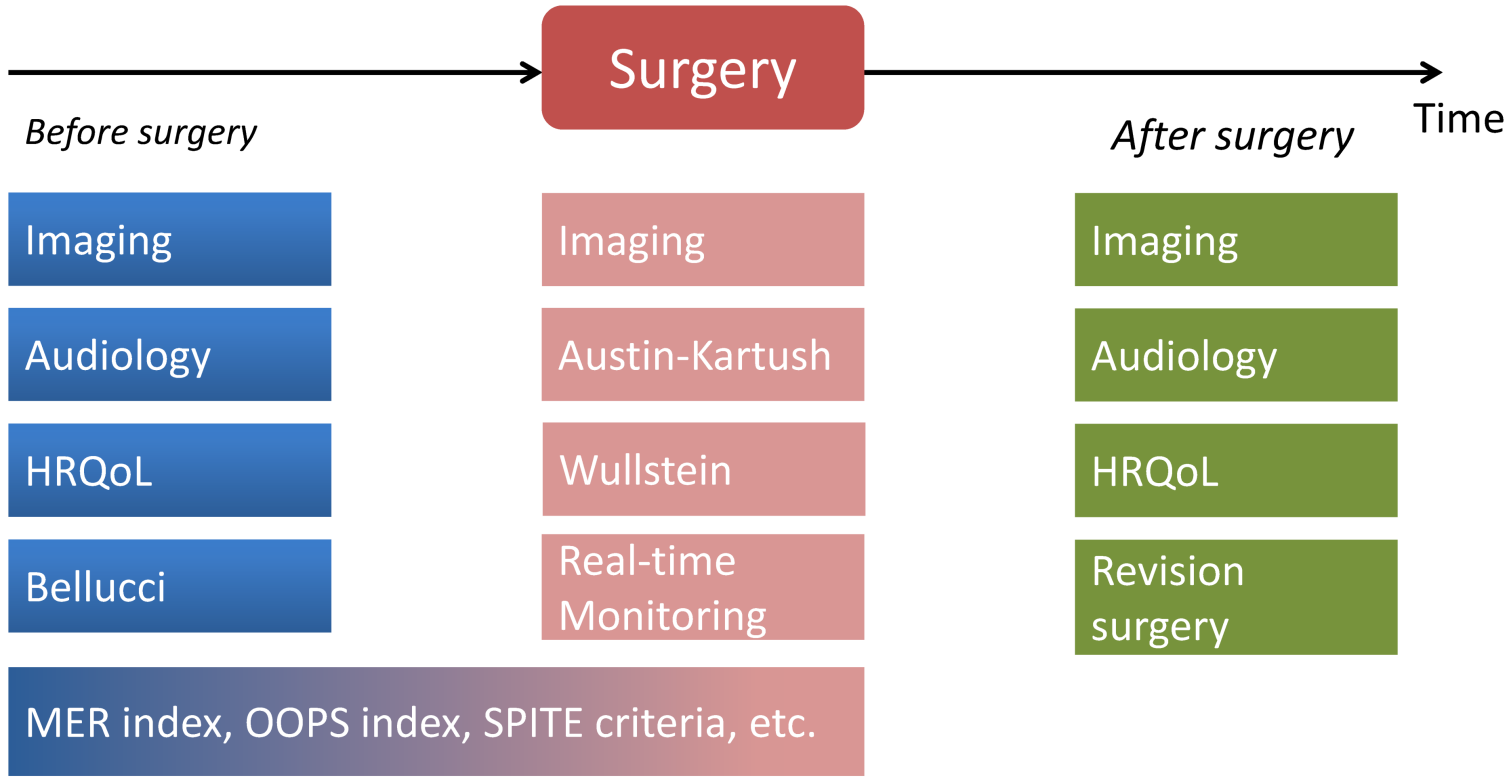
Diagnostics and assessment instruments are applied at different points and times in the treatment course. In comparison of the time course (pre- and postoperative) data related to audiology and the quality of life have a good assessment quality while imaging can be used with different questions throughout the whole process. Assessment instruments that integrate the patient’s history as well as intraoperative findings in order to evaluate the individual risk profile and the probable success, combine pre- and postoperative data. Classic instruments for the standardized, but merely intraoperative assessment and documentation are the classifications described by Wullstein [1] and Austin-Kartush [40]. The intraoperative real-time feedback is a new procedure to generate statements on the acoustic reconstruction quality.

The process chain of quality assessment for tympanoplasty already starts with the description of the preoperative situation, includes the actual surgical intervention, and ends long time after final wound healing with the clinical status at least one year after surgery (Figure 2 (Fig. 2)). It must be taken into account that the wish of possibly complete description of the status on the one hand and the clinically necessary as well as economic reality may diverge – not all parameters that may be assessed and documented, obligatorily contribute to the description of the quality. Nonetheless, relevant and high-quality clinical studies allowing valid conclusions, must not and cannot undercut a minimal standard. In particular the inhomogeneity of disease entities requiring middle ear reconstruction, makes a detailed individual stratification necessary. Otherwise the statements are difficult to be applied and the result are diluted by too many influence factors.

### 2.1 Assessment parameters of tympanoplasty

With regard to clinical as well as scientific questions, mainly functional parameters are applied internationally to assess the surgical outcome after middle ear reconstruction. Additionally, pre- and postoperatively the inflammation situation and the incidence of recurrences or the necessity of surgical revision are mentioned.

#### 2.1.1 Audiological parameters

Under functional aspects, the pure tone audiometry prevailed as the most important psycho-acoustic measurement instrument, which represents also a basis for correlation with other outcome parameters because of its confirmed validity [12]. For comparative evaluation, the difference between the air conduction threshold and the bone conduction threshold, the so-called air-bone gap (ABG) are measured before and after surgery. In this context, the calculation of the average from measured sound pressure level in defined frequencies, also called pure-tone average, is an established tool. An international definition of the frequencies that should be selected, does not exist. In the Anglo-American countries, the frequencies of 0.5, 1, 2, 3 kHz prevail based on the recommendations of the “Committee on Hearing and Equilibrium” (1995) [13] of the “American Academy of Otolaryngology – Head and Neck Surgery Foundation” (AAO–HNS). Table 1 (Tab. 1) shows a summary of the recommended parameters. The basis for calculation of the ABG must be discussed critically. The value of the ABG varies not only with the decrease of the mere sound conduction component by reduction of the air conduction threshold, but also with a modification of the bone conduction threshold before and after surgery. With constant air conduction threshold, an increase of the bone conduction threshold by 20 dB reduces also the ABG. This effect of bone conduction increase has been documented several times [14], [15], [16], but it is completely neglected in the calculation of the ABG performed according to the current recommendations. The calculation of the ABG as difference of postoperative air conduction threshold and preoperative bone conduction threshold was often applied before introduction of the recommendations of the AAO–HNS and neglects the mentioned problem [17].

Similar recommendations regarding the quality criteria and a minimal standard of outcome communication in clinical studies issued by the committee of German-speaking audiologists, neuro-otologists, and otologists (Arbeitsgemeinschaft Deutschsprachiger Audiologen, Neurootologen und Otologen, ADANO) do not exist, they are currently elaborated. In the German as well as international literature, diverging frequency ranges including 4 and 6 kHz and the entire measured frequency range are found. Speech audiometry is another audiometric procedure that is only rarely applied for description of functional results after tympanoplasty. For speech audiometry, again, no international standard exists regarding the measurement methods and the documentation of the results. The most recent recommendations of the AAO–HNO include speech audiometry as measurement procedure and request the depiction of the word recognition score (WRS) together with pure tone audiometry (PTA) in a combined graph, a so-called scattergram” [18]. Generally, the implementation of speech audiometry is plausible since the PTA only reflects the tonal hearing threshold and does not allow a statement on higher processing levels of hearing. Patients with similar postoperative outcome in the PTA may achieve completely different results in speech audiometry, for example when the hearing results after acoustic neuroma surgery are compared to those after middle ear surgery. In this context, however, it is a problem that there are no binding specifi-cations on the speech material and its presentation. The authors claim 40 dB as presented sound pressure or the maximally comfortable loudness. Regarding the selection of the speech material, at least 50 word (of the native language) should be provided by a sound source, alternatively, the presentation by the examiner as spoken “live” speech audiometry is possible. Thus, only within the defined standard, uncertain variables exist as well as differences of the basic measurements that are applied regularly in Germany. Even in the case of applying the required AAO–HNS standards, comparative statements are clearly limited. So efforts on the implementation and consequent application of international standards for outcome reporting have to be supported. Despite the fact that the AAO-HNS recommendations on the reporting standard of 1995 are currently the only ones that have been published, they are applied in the literature only to a limited extent.

A literature research identified more than 160 publications on the key word of surgery for hearing improvement between 2005 and 2015; they were evaluated regarding the application of the AAO-HNS recommendations of 1995. It could be revealed that only in 59% the PTA (hearing threshold and ABG) was given as average with standard deviation, so 41% of the trials could not be included in a meta-analysis and statistical statements are of limited value. The frequency range was described in 85% of the studies, which means that 15% of the publications did not define this parameter. The recommended test frequencies of 0.5, 1, 2, and 3 kHz were applied in 47% of the studies. Speech audiometry was only performed in 4%, while the follow-up interval to assess the outcome amounted to less than 6 months in 60% [19].

Even if the definition of the test frequencies regarding the inclusion or exclusion of 3 and 4 kHz has always been controversially discussed since the introduction of the recommendations, it cannot justify the complete lack of mentioning any frequencies in 15% of the evaluated studies. At first sight, the differences of the dB value of the PTA-ABG calculation with or without including 3 and 4 kHz seem to be marginal but the statistical analysis reveals that they are significant [20], [21]. For a comparison of the hearing results of various studies, the information about the frequency range is essential. So it is alarming since the publications appeared in established ENT-specific journals after peer-review procedure. Also the value of the study results for assessment of the clinical long-term course is clearly limited.

More than 20 years after formulating a recommendation of a minimal quality standard regarding the reporting of the outcome of hearing results, their application in the study landscape shows a high need of improvement. At the same time, it must be feared that the extended recommendations from 2012 to include speech audiometry will only be realized reluctantly, also because of the mentioned measurement difficulties. The efforts made to find a consensus in the German speaking and European ENT-specific societies regarding a minimally standardized reporting of results should be promoted vigorously. Only in that way, it may be assured in the international discourse that established and validated audiometric measurement methods and parameters (such as for example test frequencies, definitions of observation intervals, calculation bases of the ABG, a1 value, percentage of intelligibility in the free-field at 65 and 80 dB (at 65 dB in noise), maximal understanding of polysyllables, or others) will be further taken into consideration and are not sacrificed to politically motivated rapid decisions only because they had never been defined as quality parameters.

#### 2.1.2 Health-related quality of life (HRQoL)

For several years now, measurement instruments on the assessment of the health-related quality of life (HRQoL) as outcome parameter become more and more important [22], [23], [24]. This development is also observed in otology [25], [26], [27]. The HRQoL is understood as multifactorial construct covering 4 dimensions: physical complaints, psychic condition, functional impairment in daily life, and impairment of interpersonal relationships. Those dimensions are analyzed by means of targeted items from the point of view of the patients with regard to his specific disease [28]. Since every disease has different symptoms, the measurement of the HRQoL has to be based on disease-specific measurement instruments. Most of the validated HRQoL questionnaires for the field of otology are available in English. Tympanoplasty as surgical procedure applied in a heterogenic group of middle ear diseases, can only be assessed indirectly in the context of a disease-specific evaluation. However, for chronic otitis media and cholesteatoma as well as for surgical interventions the “chronic ear survey” (English and Chinese) [29], the COMQ-12 (English, Dutch) [30], [31], the “chronic otitis media outcome test 15 – COMOT 15” (German) [25], and the “Zurich chronic middle ear inventory – ZCMEI-21” (German) [27] are available. Using HRQoL measurement instruments, it must be taken into account that the questionnaires have to be validated in the according language. A questionnaire that has been developed and validated must not be simply translated by an investigator and applied without re-validation because the translation into another language may change the meaning of the single items and thus even the overall statement of the test. Nonetheless, they may be used as orientation for patient interviews. As a consequence, the selection of suitable German measurement instruments for the context of tympanoplasty, i.e. surgery for hearing improvement, after chronic otitis media is rather low. In addition, the practical orientation of the test and the weighting of the relevant dimensions may be different leading to inexactness of the assessment between the measurement instruments [27]. The hearing impairment is certainly an important influence factor of the HRQoL in chronic otitis media, but it is perceived in different ways by different patients and individually weighted [32]. The same is true for otorrhea [33], [34]. In summary, the number of items that inquire the impairments by the according symptom is decisive for the weighting and the differentiated assessment. Those gaps or specific focus of the single measurement instruments have to be identified in future studies. This is only possible by applying them in specific studies that include also the appropriate HRQoL measurement instruments beside objective audiological parameters. Only in that way, the improvement or deterioration of the quality of life assessed by the patients can be correlated reasonably with the results of audiometry.

It is basic for an implementation of the HRQoL in clinical and scientific routine to carefully develop and apply the measurement methods. Measurements of the quality of life must be sharply delimited from patient interviews that inquire about symptoms and possible impairments by means of own item lists. They can exclusively contribute to the practical documentation of complaints. In order to find scientifically sound, reliable conclusions, the application of psychometric measurement instruments that meet all quality criteria (objectivity, reliability, and validity) of a standardized measurement procedure, are essential. Beside statistical evaluation, only those allow a comparability of data and can contribute to the assessment of outcome parameters.

Currently it is still quite easy to keep an overview about the studies on surgical hearing improvement that include HRQoL data in addition to clinical and audiometric outcomes. With the continuous development of new measurement instruments for other diseases, the HRQoL data situation, also for tympanoplasty, will increase in the future.

### 2.2 Factors influencing the hearing result

Beside the actual reconstruction of the conductive apparatus, many factors influence the postoperative hearing outcome. Undoubtedly, the postoperative ventilation of the middle ear is one of the most relevant factors. The underlying middle ear pathology, its extent, the presence of residual ossicles, the status of the middle ear mucosa, and other factors additionally influence independently the postoperative acoustic middle ear function. Studies encompassing statements on the postoperative hearing outcome often do not mention those factors in detail. One can only speculate about the reasons. The stratification based on too many factors easily reduces a study population of more than 100 patients to single-digit values so that no reasonable statistical calculations can be made. On the other hand, experimental as well as clinical results of the literature confirm that some factors have a definite influence on the hearing outcome and thus they should be included in the description of the patient population and the discussion of the hearing results. The publication of results after tympanoplasty pursues three objectives: (1) the assessment of the surgical method or a reconstruction technique or prosthesis, (2) the comparability of the results with other case series and studies, and (3) the determination of the prognostic success [35]. This is expressed in the attempt to establish assessment systems that aim at describing the extent of the middle ear pathology based on the weighted assessment of influence factors and at the same time at providing a prognosis for successful tympanoplasty.

Already the classification presented by Wullstein in 1956 [1] contained a prognostication upon the postoperative hearing result beside the description of the middle ear reconstruction type. Bellucci described a dual assessment system measuring the possible success of tympanoplasty depending on the presence or tendency to middle ear infection [36]. As indication of inflammation, he took otorrhea and classified the patients into four groups (otorrhea; I: none, permanently dry; II: sometimes; III mostly to permanently humid; IV: mostly to permanently humid plus malformation of the nasopharynx/cleft palate). According to this classification, the probability for successful tympanoplasty with inflammation control decreased with higher classification. A 2-year inflammation control is reported by Bellucci in 88% (162/183) in group I; in 58% (76/135) in group II; 24% (8/33) in group III, and 8% (1/12) in group VI. Audiological data were not mentioned. Nonetheless, this first differentiated analysis of the study population allows a limited success prognosis based on defined influence parameters. Later, a classification was suggested with additional inclusion of the reconstruction types described by Wullstein (type I–V) [37].

Another instrument that was described by Black as “SPITE criteria” encompasses five dimensions, which have to be assessed by the surgeon based on a questionnaire [38]. The single items refer to the details of the surgery, the prosthetic reconstruction (prosthesis), the status of the infection, the status of the mucosa of the middle ear and the mastoid mucosa (tissue), and the middle ear ventilation (summarized as “Eustachian tube”). The number of answers having a negative impact on the result are summed up, based on the score a cluster is allotted, and finally the hearing outcome is stratified according to the risk assessment. Because of the comparably high effort of 19 items and the calculation of the cluster allotment, this actually good measurement instrument could not be generally established.

Clearly more frequently, the application of the Austin classification [39] is found in the literature, mostly in the modification of Kartush. Austin focused originally on four conditions of the ossicular chain that result from the combination of malleus handle (M) and stapes superstructure (S) [40]. Initially, he differentiated between group A: M+/S+; group B: M+/S-; group C: M–/S+, and group D M–/S– (Table 2 (Tab. 2)). Since neither stapes fixation nor malleus/incus fixation were taken into consideration, Kartush extended this classification to the Austin-Kartush classification and integrated it into the “Middle Ear Risk Index”, MER index, abbreviated MERI [41]. The MERI integrated furthermore the inflammatory status of the ear measured based on otorrhea according to the Bellucci classification. Additionally, other aspects were assessed such as the eardrum (intact/defect), the presence of cholesteatoma (yes/no), granulations or discharge (yes/no), and previous surgeries [41].

In the most recent version, “smoking” is mentioned as additional risk factor [42] (Table 3 (Tab. 3)). In comparison to non-smokers in the patient populations, smokers had a threefold higher risk for recurrent perforated eardrums after 6 months, a higher severity of the disease, and needed significantly more often canal wall down (CWD) surgery for eradication of the disease.

In contrast to other instruments, the MERI assesses the extent of different pre- and intraoperative risk factors with a numeric value. The sum of those values leads to the risk that increases with higher scores and thus allows a prognosis of the probable success of tympanoplasty. So the MERI is comparable to the modern HRQoL measurement instruments leading to a more frequent application also due to its easy handling and significance.

**Figure 3:**
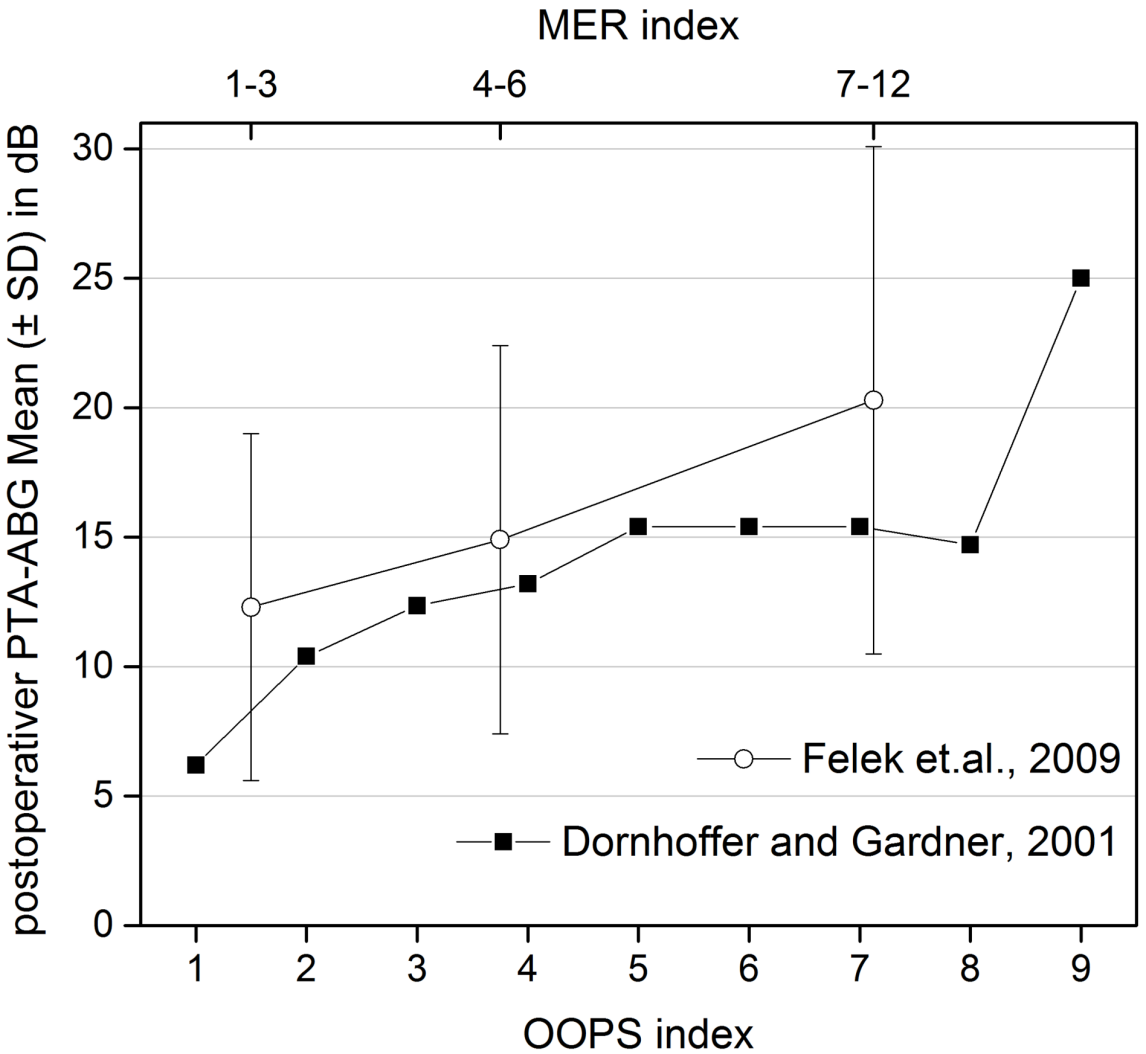
MER-OOPS: correlations of the postoperative air-bone gap (ABG) and the scores of the middle ear risk (MER) index (upper y-axis: data of dB as average value and standard deviation) according to Felek et al. (2010) [57] and the ossiculoplasty outcome parameter staging (OOPS) index (lower y axis; data of dB as average value according to Dornhoffer and Gardner in 2001 [43]. For the OOPS-index, a correlation coefficient of 0.8 is given. The correlation between the increasing scores in the indices and the remaining ABG is clearly seen.

The development of the described assessment systems was merely empirical and all considered influence factors were taken into account because of clinical observations. In 2001, Dornhoffer and Gardner were the first to perform a multivariate analysis correlating the MERI influence factors with the hearing outcome. They were able to show in 200 patients that the malleus handle, the condition of the middle ear mucosa, the surgical technique, the revision situation, and otorrhea had a good correlation with the hearing result [43]. So this study could partly rebut some aspects of the Bellucci and Austin-Kartush classifications and confirm new influence factors regarding their significance. The results were summarized in the OOPS index (ossiculoplasty outcome parameter staging index) that revealed a very good correlation with the hearing results (r=0.8) in this study. MER index and OOPS index are two assessment instruments that allow a risk and outcome prognostication based on statistically justified item assessments (Table 3 (Tab. 3), Figure 3 (Fig. 3)).

#### 2.2.1 Malleus handle

The malleus handle represents a preferred position for the coupling of middle ear prostheses [6]. Experimentally [44], [45] as well as by simulated calculations [46], [47], this hypothesis could be confirmed. A majority of clinical studies could confirm in vivo that the presence of the malleus handle was a positive predictor for a good postoperative hearing outcome [35], [43], [48], [49], [50], [51]. Even if coupling to a present malleus handle must be generally preferred, too high tension with deflection of the stapes (with intact superstructure) from its vertical axis should be avoided [4], [6]. The resulting pretension in the annular ligament causes measurable transmission losses (see chapter 4.4). The importance of the malleus handle independent from the condition of the stapes superstructure could recently be confirmed in a meta-analyses. Blom et al. [52] assessed 9 studies that allowed a comparative data-based evaluation of the Austin-Kartush classification. Hereby, group B (M+/S–) achieved an ABG of 11.1 dB (10.3–11.8 dB; 95% confidence interval (CI), n=246) which was significantly better (p<0.01) than group C (M–/S–) with 15.7 dB (14.0–16.7 dB; 95% CI, n=208) and group D (M–/S–) with 16.5 dB (15.2–17.9 dB; 95 CI, n=157). In summary of the experimental and clinical results, this confirms the crucial role of the malleus handle as significant prognostic factor for the postoperative hearing result.

#### 2.2.2 Middle ear mucosa

Pathological changes of the middle ear mucosa may develop in the context of chronic inflammatory processes or after the mucosal trauma of middle ear surgery. Morphological changes such as granulations, fibrosis, scars, tympanosclerotic plaque, or bone formations are possible [53], [54], [55]. Those influence the gas exchange and thus the postoperative hearing result. Also dislocations of the prostheses or fixations caused by extensive mucosal reactions are frequently observed which is reflected in the hearing test results. When the mucosa was intraoperatively assessed as normal, this was a positive prognostic factor for the hearing outcome [35], [38], [43], [56]. The poorest results were observed in the studies when the mucosa was described as inflamed and fibrosed. Even if a reliable therapy of the middle ear mucosa is not available, it is helpful to know after surgery in the patient consultation and regarding the expectations of the surgeon, that statistical ABGs of 18.0±9.7 dB [43] up to 24.7±14.1 dB [35] can be expected in cases of inflammatory mucosal situations.

#### 2.2.3 First vs. revision surgery

The necessity to perform revision surgery seems to be obvious as negative influence factor on the postoperative hearing ability and is confirmed by numerous studies [35], [43], [50], [51], [56], [57], [58], [59], [60], [61], [62], [63]. Some other studies cannot confirm this effect [35], [38], [64], [65]. Hereby it must be taken into consideration that the term “revision surgery” has to be exactly defined. If all further surgeries are summarized in this term, revision interventions because of residual or recurrent cholesteatoma or persistent chronic inflammatory activity are included as well as planned second-look surgeries, interventions because of prosthesis dislocation or a small residual eardrum defect. An exact differentiation also of the surgical strategy to perform the disease eradication and the ossiculoplasty in two sessions (staged operation) must be made. Hereby, a second intervention would not be a revision surgery in the proper sense and not be considered as negative predictor. The terms of “revision” and “staged” do not contain a statement on the clinical indication or surgical details and thus do not allow a statement on the origin of the clinical necessity. This definition-based inexactness is also considered as flaw in the current AAO–HNS guidelines and as the responsible factor for contradictions in the literature [17]. In view of the multitude of articles that describe the negative influence of the parameter “revision surgery”, the indication for revision seems to be disease-related in the majority of the studies.

The intention and advantages of the risk assessment of tympanoplasty are not only a differentiated counseling and better information of the patients. They are extremely important for a differentiated description of a study population and comparative evaluation of study results. They oblige the authors to prospectively determine and thus exactly define the influence parameters that are found in their patient population, which is difficult or impossible to realize in a retrospective study design, on which most of the articles are based. A consequent application of the assessment instruments in the context of clinical studies would be desirable because the patient population is similar in the therapeutic endpoint with the term of “tympanoplasty” but it comprises different disease entities with different prognostic starting points [17]. For this purpose, the current version of the MERI [42] and the OOPS index [43] are good tools.

## 3 Quality control in reconstructive middle ear surgery

During and after a tympanoplasty, the surgeon has only limited possibilities to assess the quality of the reconstruction he has performed or to identify the reasons for failure. For the intraoperative assessment and improvement of the acoustic quality of reconstruction, several solutions were described that are different with regard to their practicability and importance. A measurement system that consists of a combination of electromagnetic stimulation and laser vibratiometry, allows a fine tuning of the reconstruction in real-time and seems to be very promising. To assess an unclear postoperatively residual conductive hearing loss, imaging procedures to identify the origin can be successfully applied.

### 3.1 Intraoperative assessment of the reconstruction quality 

The wish to retrieve information about the expectable hearing improvement already during surgery, is understandable. If ossiculoplasty is performed under local anesthesia, the patient himself can give direct feedback about the acoustic result of the ossicular reconstruction. Further manipulation of the prosthesis such as change of the coupling, length or position between the ossicular remnants or the reconstructed eardrum may lead to a subjectively optimal outcome for the patient. In case of surgeries performed under general anesthesia, this possibility is not available. The surgeon must then rely on his experience and knowledge about the biomechanics of the middle ear.

In order to add an objective reconstruction quality assessment to those subjective factors, several strategies were pursued. The starting position for its implementation is rather unfavorable. The stimulation of the prosthetic and ossicular vibration must be possible with a defect or reconstructed eardrum and at the same time meet the requirements of sterile precautions of the surgical intervention. The acoustic stimulation via insert phones can be realized in a sterile way with some effort, but acoustic stimulation is not possible with a defect or forward-folded eardrum. Even the selection of the measurement system and parameters determining the quality of the reconstruction is subject to narrow basic conditions. The measurement system should react without time delay and thus display changes directly – in real-time – at the reconstruction. Time-consuming measurements of acoustically evoked potentials are not suitable.

The combination of a mechanical stimulation and an optical measurement (LDV) of the ossicular vibration seems to be most appropriate for the requirement of real-time monitoring. The quality of the coupling of stapes prostheses could be successfully investigated with such an experimental measurement system [66].

Other measurement systems and methods with acoustic stimulation [67], [68] or mechanical stimulation via the floating mass transducer (FMT) of the Vibrant Soundbridge^®^ [69], [70], [71] are clinically less suitable.

### 3.2 Real-time feedback and ossiculoplasty monitoring

The mechanical stimulation of the ossicular chain, which is independent from the status of the eardrum, uses a method of hearing aid development where a magnet is placed on the umbo and driven electromagnetically by a coil [72], [73], [74], [75], [76]. Even experimentally, this type of stimulation was described as alternative to acoustic stimulation [66], [77], [78], [79]. In a recent study, this procedure, as combination of electromagnetic stimulation and LDV measurement on the footplate, could be applied intraoperatively for quality control of tympanoplasty [80]. 

**Figure 4:**
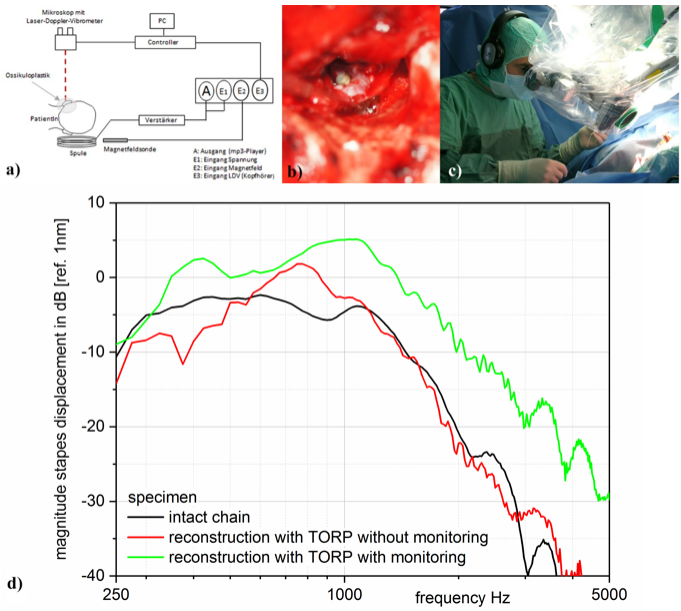
Display of the real-time feedback for ossiculoplasty. a schematic measurement setup; b magnet (*) on the umbo/eardrum; c surgeon with earphones; d improved middle ear transfer function by acoustic feedback. Via the coil that is placed under the patient’s head the magnet is set in vibration on the umbo/eardrum after performed ossiculoplasty. This is transmitted on the stapes and registered on the footplate by means of laser-Doppler-vibrometer (LDV). As stimulation signal of the coil, music can be played that is forwarded via the ossicular reconstruction to the footplate and registered by the surgeon via earphones. In real-time, changes of the prosthesis position and coupling can be “heard” and an optimal reconstruction result can be achieved [80].

It must be taken into account that the best hearing result that can be achieved by means of real-time feedback can only be identified by comparative transmission measurements. The wish to discover a technically exact and generally defined transmission function of the middle ear must remain unfulfilled because sound transmission through the middle ear corresponds to an individual characteristic curve. Under stimulation of the eardrum with 94 dB SPL, the frequency-specific ossicular vibration (measured on the stapes footplate) varies of ±10 dB, which corresponds to a corridor of the transfer function of 20 dB [81]. In other words this means that the middle ear transmission function of two regularly hearing individuals may vary of up to 20 dB. If an objective statement had to be given intraoperatively about the quality of ossiculoplasty, it is not possible to refer to a reference curve. It would be possible to define the corridor of 20 dB of the transmission function, but for the individual ossicular reconstruction the change of the transmission function during manipulation is more relevant. The reconstruction quality is shown to the surgeon optically (as transmission curve) and/or acoustically (as acoustic signal via an earphone) and can be changed by manipulating the prosthesis while the changes are displayed in real-time (real-time feedback). In this way, a fine tuning by changing the prosthesis’ position and coupling can be performed (Figure 4 (Fig. 4)). First clinical applications could confirm the general intraoperative use of the system and show that the transmission properties of a reconstruction by means of feedback are improved by up to 25 dB [80]. Further studies are currently performed to assess the effect on the postoperative hearing outcome in the long-term course.

### 3.3 Postoperative quality control by means of imaging

After surgery, audiometric data such as tone audiometry with determination of the postoperative ABG, absolute and as difference regarding the preoperative original findings, are taken as standard reference (see chapter 2.1). Significantly more rarely, speech audiometry is applied. Additionally clinical data on the status of the eardrum (intact/defect), sometimes the ventilation (Valsalva), and the inflammation are assessed, unfortunately not in a standardized way and thus they are individual.

The evaluation of a postoperatively remaining conductive hearing loss is often problematic in cases of closed and/or reconstructed eardrum because a prosthetic reconstruction cannot be assessed visually. Generally, beside the complete dislocation of the prosthesis with complete transmission loss, also partial dislocation and changes of the coupling at the prosthesis-ossicle-interface are possible reasons. Of course, other reasons may also be disease-related, biological, and thus independent from the prosthesis, factors that cannot be influenced by the surgeons. The gold standard to find the origin and if possible a causal therapy is the surgical exploration [82], [83]. Besides, the value of imaging procedures could be investigated in the context of studies. In contrast to the preoperative imaging of the middle and inner ear, for which CT scans prevail as gold standard, its significance after surgery is limited because of the artefacts of metallic middle ear prostheses. This aspect has to be considered when evaluating a series of studies that describe computed tomography as method of choice for postoperative assessment of middle ear prostheses [84], [85], [86], [87], [88]. In those studies, exclusively prostheses made of hydroxyl apatite had been used so that no artefacts were displayed in the CT scans. With this background of the increasing application of titanium prostheses, this aspect must be particularly observed [89], [90], [91]. Rotational tomography (RT) and cone beam tomography (CBT) are non-invasive imaging procedures that can be well used for postoperative assessment. The advantages of conventional CT technique are avoided (20–60 mGy radiation exposure, artefacts, partial volume effect) [92]. Displaying the ossicles and titanium prostheses is possibly by means of RT with high resolution capacity [93] and at the same time clearly less radiation exposure (10–15 mGy). Also CBT provides a raw method-related dataset that can be evaluated with the suitable software nearly free of artefacts according to different questions. The radiation exposure amounts to 10% of a CT scan of the temporal bone or is twice as high as conventional radiography. 

**Figure 5:**
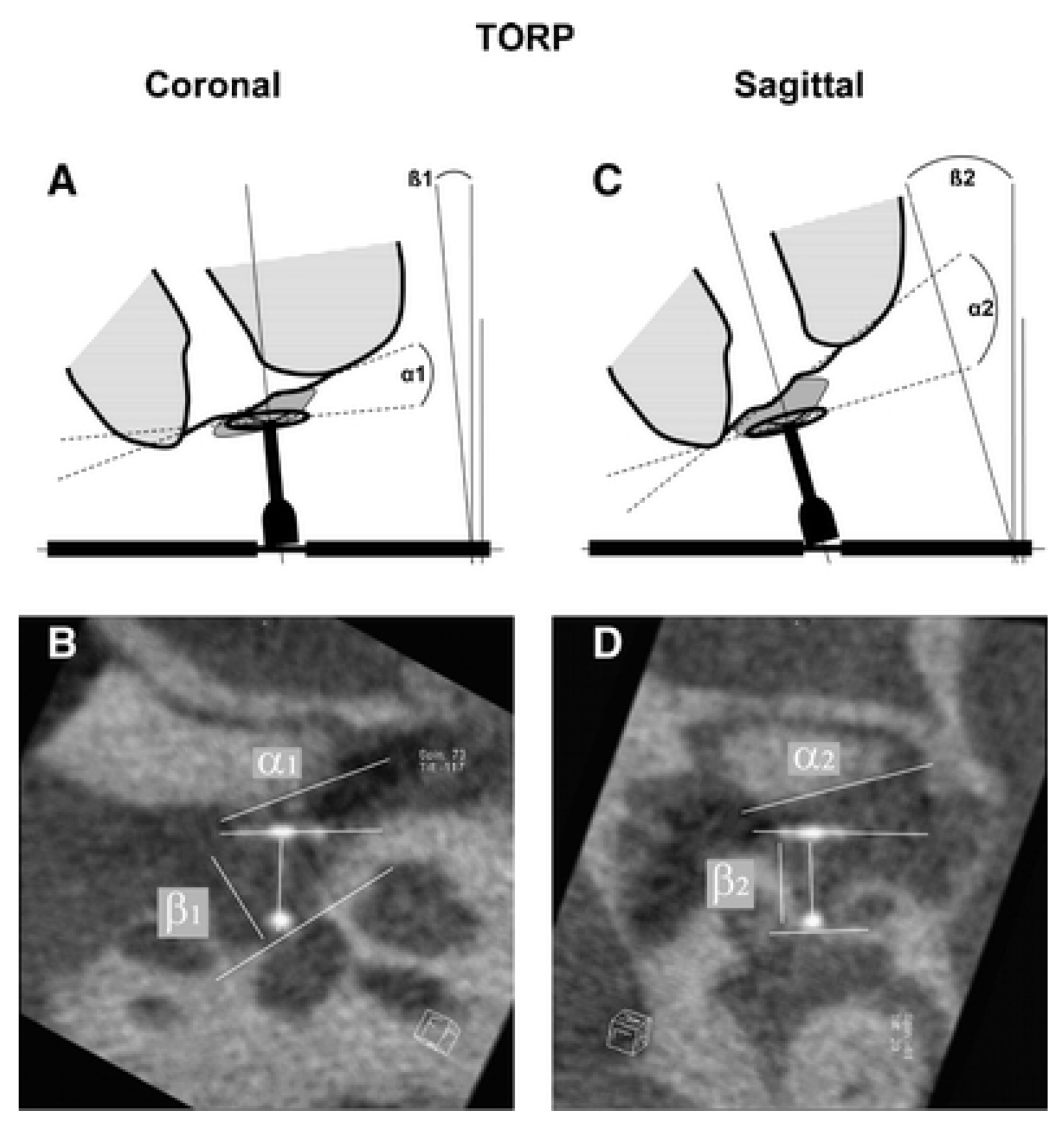
Imaging for postoperative quality control. In order to assess the position of the prosthesis in the middle ear by means of rotational tomography (here: total prosthesis, total ossicular replacement prosthesis (TORP)) the coupling angle a between the prosthesis head plate and the eardrum/manubrium and the angular deviation ß of the prosthesis stem from the vertical stapes axis is determined in coronal section (a; b) and sagittal section (courtesy of Zaoui et al., 2014 [95]).

**Figure 6:**
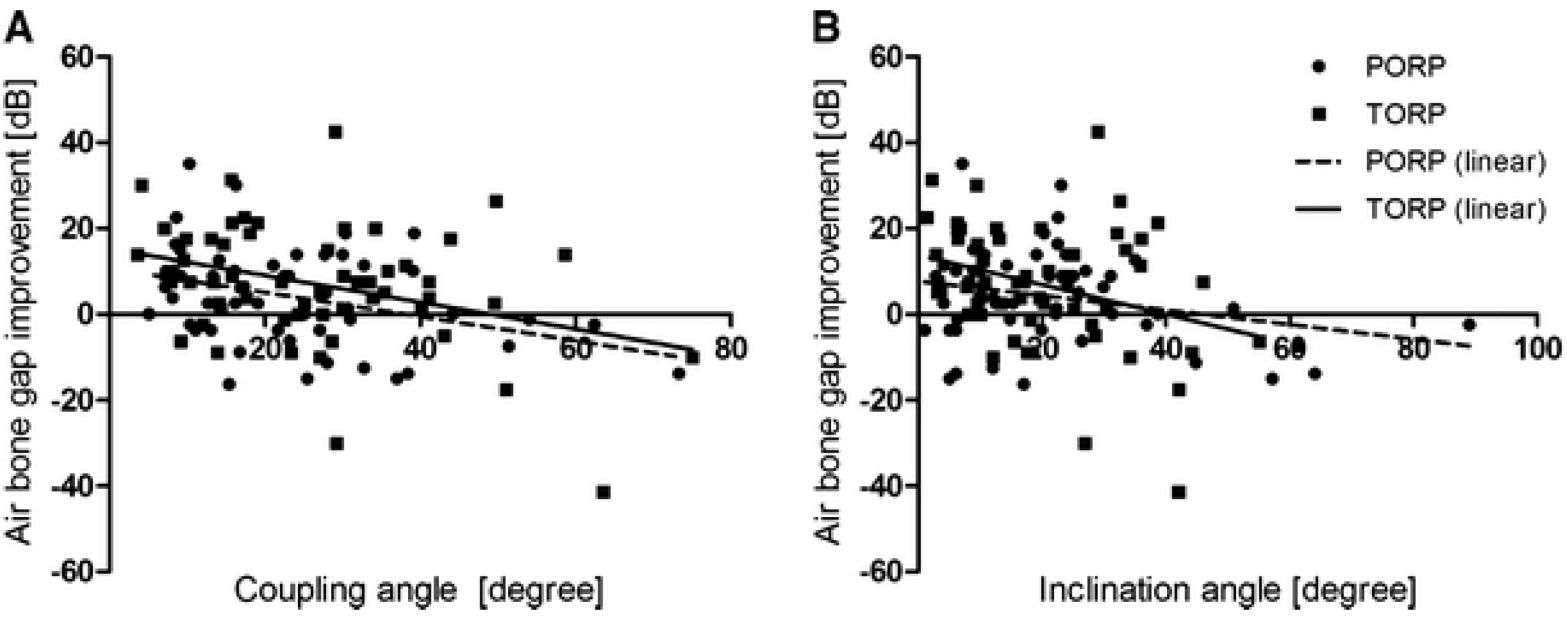
Correlation of the coupling angle and hearing outcome. Correlations between the coupling angle a (a) and the inclination angle ß (b) (measured in °) and the postoperative air-bone gap (ABG) improvement. The figure displays all prostheses examined in the study, 52 PORP and 55 TORP. (TORP: total ossicular replacement prosthesis; PORP: partial ossicular replacement prosthesis) (courtesy of Zaoui et al., 2014 [95]).

Experimentally, RT and CBT provided statements about coupling and the spatial orientation of titanium prostheses [94]. After indirect contrasting of the eardrum with sponges that had been impregnated with a contrast agent, the angle between the prosthesis plate and the eardrum level could be determined as well as the angle that the prosthesis stem had in relation to the stapes vertical axis. Furthermore, experimentally statements were possible regarding the positioning of a TORP foot on the stapes footplate. Interestingly, those statements do not only answer the question of postoperative dislocation of the prosthesis as possible origin of a residual conductive hearing loss, but also allow drawing conclusions regarding the biomechanical conditions of sound transmission. The direct comparison of imaging and functional transmission measurements by means of LDV shows a tendency that the transmission curve decreases with increasing angular deviation of the prosthesis stem from the vertical ideal line of the stapes [92]. This correlation observed between the coupling angles and postoperative hearing result could be confirmed in a subsequent clinical trial. For PORP and TORP, significant correlations were found between the postoperative ABG and the prosthesis-ossicle angle or the eardrum-prosthesis plate angle. Taking into consideration all prostheses (PORP and TORP), the biomechanical relationship between the position of the prosthesis and the angular deviation from the stapes vertical axis was confirmed (Figure 5 (Fig. 5) and Figure 6 (Fig. 6)) [4], [44]. Based on calculations, the transmission loss increases with the square of the cosine of the actual angle to the vertical, which means a loss of 6 dB for an angular deviation of 45°. Evaluating the results after partial and total prostheses in a differentiated way, a significant correlation of ABG and inclination angle is revealed only for the TORP. For PORP, only the coupling angle of the eardrum and the prosthesis plate correlates with an improvement of the ABG. Additionally, single cases were identified for which already imaging could diagnose a dislocation of the prosthesis [95]. Hereby it was possible to visualize dislocations between the eardrum and the prosthesis as well as between the stapes head or stapes footplate. The postoperative imaging by means of RT can be successfully applied for postoperative quality control. Beside differentiated statements about the position of the prosthesis and the coupling, also functional evaluations of the transmission and thus the postoperative ABG are possible. From a scientific point of view, especially in an experimental setting, this procedure may identify the exact position of the prosthesis in the temporal bone specimen. Regarding the multitude of trials for middle ear reconstruction, they are often not quantified because of the enormous effort. Nonetheless, the determination of both parameters, coupling and inclination angle, provides the possibility to experimentally evaluate the middle ear transmission curve more exactly and to make prognoses to a certain extent.

## 4 Development of functional elements for middle ear prostheses

Beside sound transmission, the middle ear has the function of adjusting static pressure variations. The middle ear has 2 working ranges for which it is functionally equipped: the nano-area for sound transmission for hearing and the macro-area for the compensation of atmospheric pressure changes. Regarding the compensation of atmospheric pressure changes, the eardrum, the ligaments and the joints of the ossicles, and the annular ligament play a decisive role. After prosthetic middle ear reconstruction, those elements partly do no longer exist. Because of favorable properties of the material, titanium prevailed among the alloplastic materials during the last years. Beside the excellent acoustic transmission characteristics and a very good biocompatibility, the possibility to develop particularly filigree design, is of major significance. This property allows integrating functional elements in the middle ear prostheses. The aim of such functional elements is the compensation of pressure variations [96]. Generally, functional elements may be integrated at the prosthesis plate, the prosthesis strut, and the prosthesis foot.

### 4.1 Malleus (manubrium) prostheses

The extraordinary significance of the malleus handle (manubrium) for the postoperative hearing results was already mentioned in chapter 2.2.1. Efforts have been undertaken to reconstruct a completely destroyed manubrium. Several approaches to replace the manubrium, have been described [97], [98], [99], [100]. In contrast to other approaches that have been described, the Malleus Replacement Prosthesis (MRP, Kurz Company, Dusslingen, Germany) uses a titanium malleus of 0.8 mm thickness that is fixed with two 0.3 mm anchors in the bony frame of the auditory canal. This procedure minimizes the risk of extrusion of the implant or dislocation. The integration of the neo-malleus has the advantage of better PORP or TORP coupling at the tympanic membrane and thus to minimize the risk of dislocation. Furthermore, the reception of sound pressure and the transmission to the reconstructed chain with a neo-malleus is clearly superior to the reconstruction of the eardrum alone (Austin-Kartush type C).

**Figure 7:**
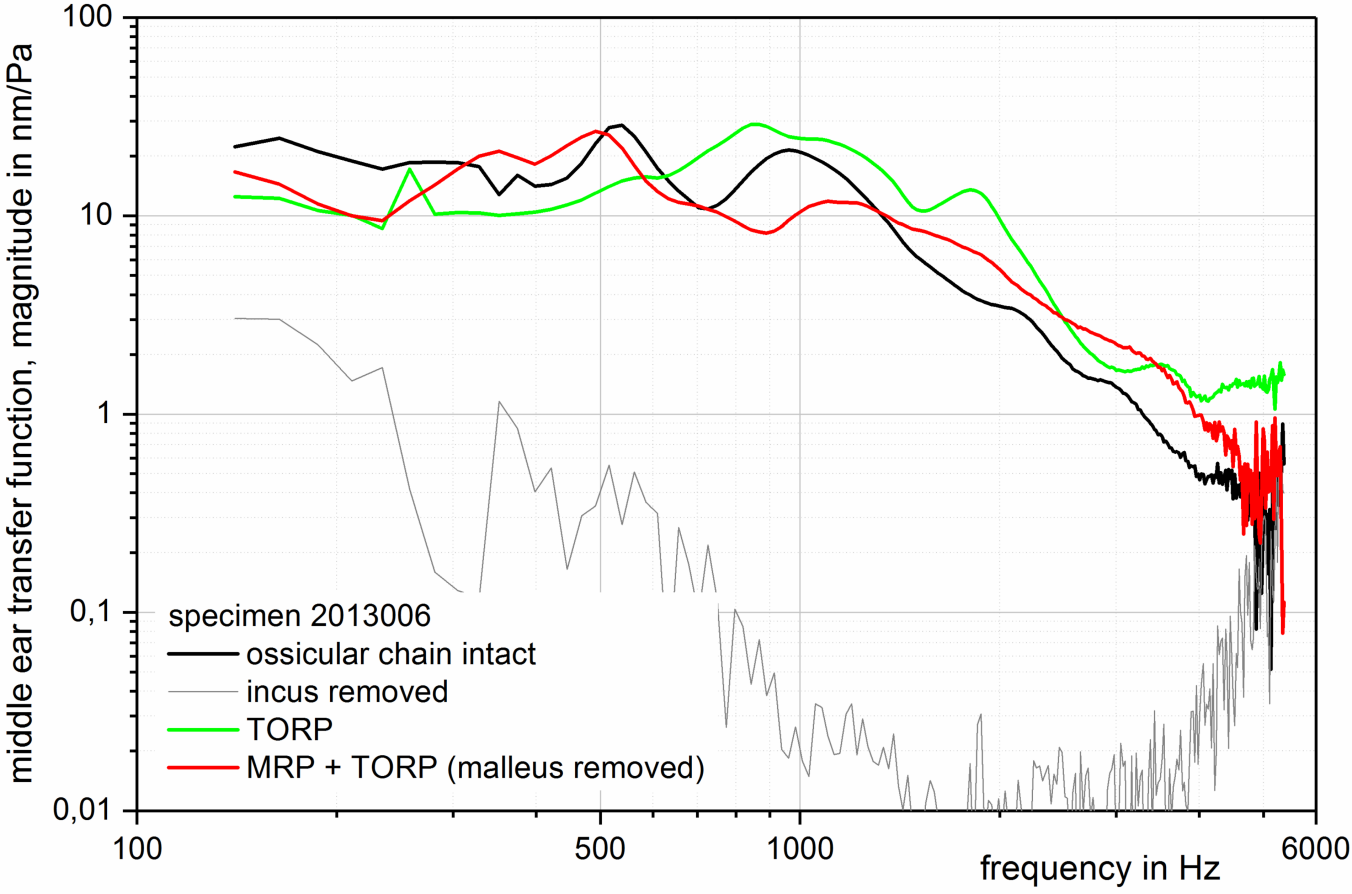
MRP-TORP. Middle ear transmission function measured in fresh temporal bone specimens. The transmission function for the total prosthesis (TORP, green line) coupled to the normal manubrium and the total prosthesis coupled to the manubrium prosthesis (MRP + TORP, removed manubrium, red line) show nearly identical results as for the intact ossicular chain (black line); measurement by means of laser-Doppler-vibrometry on the footplate; stimulation of 94 dB SPL, intact eardrum.

Experimental measurements with temporal bone specimens reveal that there is nearly no difference of the middle ear transfer function between a malleus-TORP-footplate reconstruction and a MRP-TORP-footplate reconstruction (Figure 7 (Fig. 7)). In the clinical setting, even a better result with the malleus prosthesis was achieved due to the additional influence factors. While the postoperative ABG amounted to 23.3±16.7 dB (n=27) 3 months after eardrum-TORP reconstruction, it was significantly lower with 12.5±11.9 dB (n=43) after application of the MRP (MRP-TORP reconstruction) (p=0.002) [100]. Due to the material properties, the neo-malleus anchored in the bone can be easily adjusted to the projection axis of the footplate so that extreme angular TORP deviations are avoided. An additional stabilization of total prostheses is possible with corresponding centering device on the footplate. The titanium neo-malleus can also be used as alternative coupling point for a malleo-vestibulopexy [101]. 

### 4.2 Head plate

In order to avoid prosthesis head plate dislocation from the tympanic membrane or to achieve long-term stability in this area without negative effects on the acoustic transmission properties, modifications of the rigid connection between the prosthesis strut and the head plate or the head plate itself are useful. A modification of the head plate to which 0.33 mm high spikes are fixed in direction of the eardrum, revealed experimentally a clear stabilization against lateral displacement forces [102]. Under experimental and clinical conditions, a protection could be achieved with regard to lateral dislocation of the prosthesis plate during reconstruction of the tympanic membrane. Another advantage of such an anchoring of the head plate in the reconstructed eardrum is observed especially in the early phase during or directly after surgery and wound healing. After complete wound healing, the head plate is generally fixed with connective tissue so that sufficient stability is assured.

The eardrum-implant interface also requires an optimized middle ear prosthesis because of the anatomical and dynamical circumstances. The angle between the reconstructed eardrum and the vertical stapes axis rarely amounts to the 90° that are manufactured between the prosthesis stem and the head plate. Positioning of the head plate with joints to the prosthesis stem may allow an adjustment to the uneven position of the (reconstructed) eardrum that varies according to the angle [96], [103]. In this way, the head plate passively follows the forces that postoperatively and in the context of wound healing lead to a changed position of the eardrum. Furthermore, this has a positive effect on short-term variations of the air pressure as well as longer-lasting pressure changes that occur in chronically sick ears due to changes of the middle ear mucosa. Especially in cases of chronic ventilation disorders, the position of the head plate at variable angles contributes to avoiding migrations of the prosthesis plate. Without increasing the pre-tension, an acoustically favorable coupling to the manubrium can be performed. Experimental trials with titanium prostheses with prostheses heads installed in ball-joints could show that under physiological conditions no material abrasions occur in the area of the titanium ball-joint and the transmission properties are comparable to those of the intact ossicular chain [103]. Also for total prostheses, the integration of a ball-joint is described with similar transmission characteristics [104]. The experimentally identified properties could be confirmed in first clinical studies in 60 patients [105], in 18 patients [103] for partial prostheses, and in 12 patients [104] for total prostheses. Regarding the use of total prostheses, the mobile head plate represents an additional surgical challenge that must not be underestimated because the angular variability makes a stable intraoperative positioning very difficult. So especially for those cases, centering devices in the area of the footplate are available such as connective tissue, a cartilage shoe [106], or an omega-connector^®^ [107]. Long-term results for both types of prostheses must still be evaluated. In summary, the integration of a titanium ball-joint between the head plate and the prosthesis strut are a promising innovation for compensating short-term pressure fluctuations and long-lasting pressure differences without having a negative effect on the acoustic transmission properties of the prostheses.

### 4.3 Prosthesis strut

**Figure 8:**
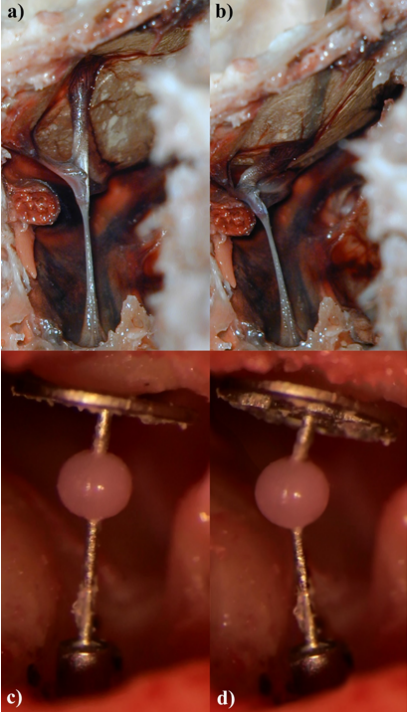
Bird’s columella and bended prosthesis. Direct comparison of an ostrich’s middle ear a and b with a bended TORP c and d under conditions of pressure balance a and c or a positive pressure in the auditory canal (negative pressure in the tympanic cavity) b and d. The movement of the prosthesis stem reduces the stress of ring ligament and footplate. When the pressure decreases, the original position is restored in both cases. So the bended prosthesis does not only represent the reconstruction of the sound conduction apparatus, but also to a certain extent the pressure balance of the intact ossicular chain (figures A and B: courtesy of Beleites et al. 2007 [108]).

The integration of a joint in the prosthesis strut is another possibility to avoid the rigid coupling between the eardrum and the footplate for compensation of atmospheric pressure variations and thus protection of the inner ear and of dislocations [96], [108]. The static prosthesis stem, following the biological example of the joint-like columella of birds, is replaced by a resilient joint. The sound conduction apparatus of the bird’s ear consists of a bony columella and a cartilaginous extra-columella that extends the eardrum in outward direction. In cases of atmospheric pressure changes, the extra-columella is shifted to the inside with the eardrum and thus protects the inner ear from pressure-related damage (Figure 8 (Fig. 8)) [108], [109], [110].

Experimental investigations with titanium total prostheses with a silicone-enhanced micro-joint that was integrated in the prosthesis stem, the feasibility of this approach could be confirmed. Bionically following the example of the birds’ columella, the prosthesis stem deviates from the vertical axis in cases of static pressure increase. The integration of a joint in a silicone mantle provides the desired reset effect so that a decrease of the pressure leads to a restoration of the original position. In this way, compared to the articulated connections in the area of the prosthesis plate that have a ball-joint with friction, there is a significant advantage: resilient joint with reset effect. There is no difference in transmission properties of the bended prosthesis in the dynamic range of sound transmission compared to rigid TORP. Under increased pressure (negative pressure in the middle ear) the transmission can be compared to the one of the intact middle ear. Currently, the silicone enclosure of the joint represents a manufacturer-related obstacle. With regard to the acoustic and static performance, however, this approach is very promising [111].

### 4.4 Prosthesis foot

The medial interface between the implant and the residual ossicles plays a particular role in many regards. On the one hand, a stable anchoring is desirable to avoid a dislocation of the total prosthesis. On the other hand, anchoring at variable angles are useful that allow positioning of the total prosthesis to the eardrum without increasing the pre-tension of the reconstruction. Furthermore, because of the punctual load of the stapes footplate after implantation of a total prosthesis, a possible fracture must be expected after intensive pressure increase [112]. A distribution of the pressure on a larger surface has to be pursued with regard to stability and protection; it is available with the omega-connector^®^ [107]. As a separate footplate prosthesis, the omega-connector^®^ provides a clearly larger surface of 1.12 mm² compared to the corresponding contact surface of titanium prostheses with 0.5 mm². Additionally, the central joint-ball represents a coupling point at variable angles for total prostheses.

This approach takes up a strategy described in 1987 suggesting a modular prosthesis concept with a surgical procedure in two sessions. A stable anchor point should be established on the footplate by incorporating bioactive ceramic on the footplate (without prosthesis-related relative movements), in a second step the proper prosthetic reconstruction of the sound conduction apparatus should be performed with a total prosthesis [113]. The use of the omega connector represents the realization of the modular prosthesis concept, however it does not resolve the problem of a bone-like coupling point on the footplate. Even if the larger surface of the omega connector provides a higher stability on the footplate and thus the risk of dislocation is lower, relative movements may lift the footplate prosthesis. So also this dislocation protection is rather a centering device. The remaining risk of dislocation might only be completely avoided by a bone-stable anchoring in the sense of osseo-integration of prosthetic material on the stapes footplate [114]. According to biomechanical calculations, this could be possible [115]. Even experimentally, a growth-factor mediated osseo-integration on the footplate could be realized in mammalian organisms, for the clinical application, however, this concept is currently not available, also because of economic reasons [116]. 

### 4.5 Influence and properties of the annular ligament

All before-mentioned functional elements are used for reconstructing the function of pressure balance of the ossicular chain. The acoustic rehabilitation alone can be well implemented with a stable and firm connection between the eardrum and the stapes under conditions of pressure balance. In order to minimize the risk of prosthesis dislocation, especially of TORP, the use of prostheses is recommended that are longer than required by the anatomical circumstances. A slightly too high prosthesis construction leads to a tight embracing and to prosthesis fixation between the stapes and reconstructed eardrum. This dynamic bracing intends to counteract the risks and forces of dislocation occurring immediately after surgery as well as in the context of wound healing [3]. In this way, outward movements of the tympanic membrane may be compensated by the fixation of the reconstruction. At the same time, the “too long” prosthesis construction leads to pretension of the remaining elastic elements: the eardrum on the lateral side and the annular ligament on the medial side. The footplate displacement in direction of the vestibulum leads to tension of the annular ligament and its resulting stiffening leads to reduced vibration and thus to a reduced middle ear transmission. This relationship between desired dynamic fixation of a prosthesis in the ossicular chain on the one hand and on the other hand the resulting reduction of sound transmission through the middle ear has already been investigated experimentally. Trials performed with temporal bones at which reconstructions were performed with prostheses of different lengths, could reveal differences in the middle ear transfer function [44], [45], [117], [118]. However, technically it was only possible to insert the prostheses in a “loose”, “well fitting”, or “tight” way according to the subjective perception and to measure the resulting transmission properties afterwards. All authors found that a “loose”, i.e. just stabilized prosthesis with low pretension provided the best transmission outcome. With increasing prosthesis length and higher fixation, first a minimal increase of the transfer function in high frequencies was observed that was associated with a decrease of the lower frequencies. With further increasing tension, a general decline of the transmission was noted. Those basic results were mainly measured with conventional differences of the length of 0.5 mm between the evaluated prostheses as they are relevant for the clinically operative routine of a surgeon. For a long time it remained unclear to what extent the pretension caused by too long prosthesis led to the stiffening of the eardrum or the annular ligament. So it was not possible to quantify the transmission loss that is generated by the fixation of the annular ligament alone.

**Figure 9:**
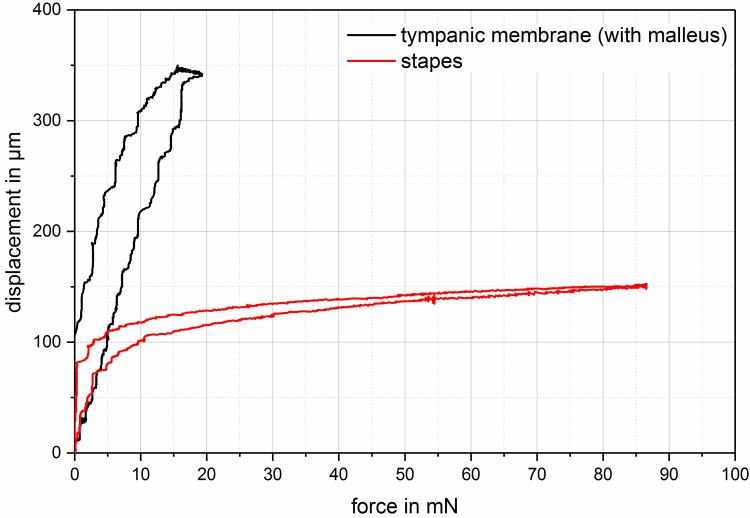
Force-displacement relation for the stapes footplate (red) and the tympanic membrane (black) in case of axial displacement in direction of the vestibulum. Because of the visco-elastic properties, the behavior for loading and releasing is different which explains the hysteresis curves. It is obvious that the annular ligament stiffens already in case of low forces and allows only little deflection. The eardrum, however, shows a nearly linear course with constant stiffness in the examined area.

**Figure 10:**
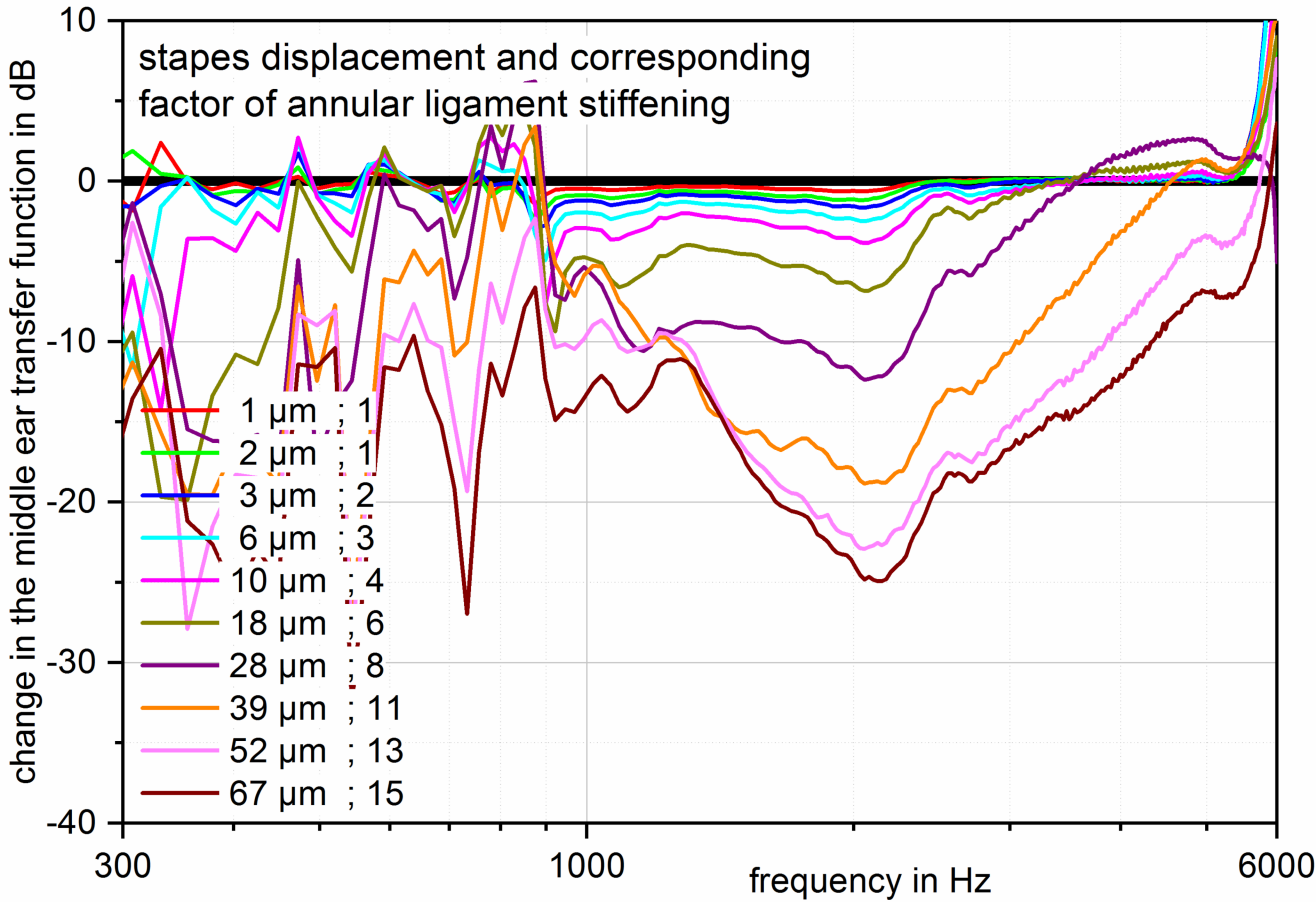
Axial loading of the stapes. Loss of the middle ear transmission function in dB, related to the neutral position of the stapes (zero line) with increasing deflection in direction of the vestibulum. The graph displays the deflection in µm and the resulting factor of annular ligament stiffening (referring to the neutral position). In case of deflection of 67 µm, stiffening of the annular ligament by factor 15 resulted in this measurement with a transmission loss of 25 dB at 2 kHz. Already low pretensions of the annular ligament (for example by longer prostheses) lead to measurable transmission losses (measurements performed in fresh temporal bone specimens, stimulation with 50 mV (corresponding to 94 dB SPL) via the floating mass transducer on the stapes head).

**Figure 11:**
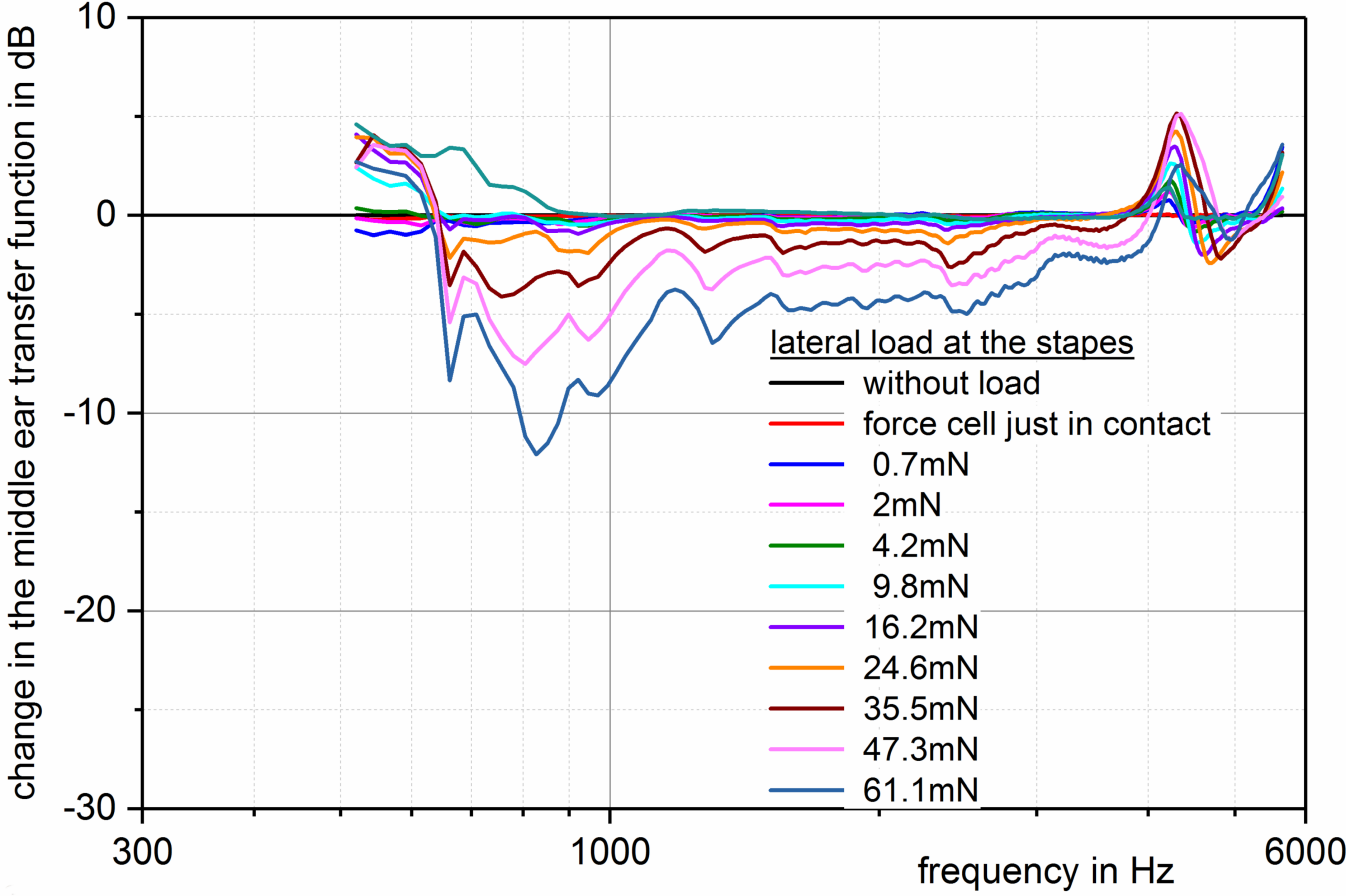
Loss of the middle ear transmission function in dB related to the neutral position of the stapes (zero line) and increasing deflection in direction of the promontorium (tilting around the longitudinal axis of the footplate). The deflection forces are measured in mN. Also tilting alone without additional displacement in direction of the vestibulum reveals transmission losses. They are clearly lower compared to axial forces (see figure 10) (measurement in fresh temporal bone specimens, stimulation with 50 mV (corresponding to 94 dB SPL) via the floating mass transducer on the stapes head).

Recent investigations of temporal bones with a prosthesis of variable lengths and at the same time determination of the stiffness of the tympanic membrane and the annular ligament could show that in deep frequencies the decrease of the acoustic transmission properties of up to 25 dB was already observed in smallest modifications of the prostheses lengths of 50 to 200 µm. Even if an elongation of the prosthesis is seen in the displacement of the eardrum at about 80% and only at 20% in a displacement of the stapes in direction of the vestibulum, this leads to an important increase of the annular ligament’s stiffness that causes the observed transmission losses. This is due to the fact that the visco-elastic properties of the eardrum show a linear behavior with a constantly low stiffness. So if the distance between the eardrum and the footplate is longer, the tympanic membrane yields with constantly low stiffness over longer distances (Figure 9 (Fig. 9)). In contrast, the annular ligament does not behave linearly [119], [120] which leads to a significantly increased stiffness already in cases of minimal displacements of the footplate (Figure 10 (Fig. 10)). In cases of deflection of 31 µm, stiffness of up to the nine-fold of the annular ligament without tension was identified with transmission losses of up to 25 dB [121]. The pretension of the annular ligament has also an impact on the transmission properties when the stapes is tilted of the vertical axis of the footplate (Figure 11 (Fig. 11)). This mechanism may also be responsible for poorer transmission properties of partial prostheses. If they are firmly fixed on the stapes, the forces that affect the head plate are transmitted on the annular ligament. In cases of total prosthesis, only a low moment of force, if at all, is generated at the contact point between the prosthesis foot and the stapes footplate. However, because of the missing anchoring in this area, this is not transmitted to the footplate. Even the use of centering devices does not change this situation so that tilting of the footplate with resulting pretension of the annular ligament does not occur [115].

It must be taken into account that the before-mentioned investigations were performed in human cadaver specimens. So biological remodeling processes as they are observed in vivo, cannot be displayed. It is still unclear how a tightly pre-tensioned annular ligament behaves after several months, if it leads to remodeling of the collagenous fiber structure and perhaps even to the development of a new “neutral position” with reduction of the pre-tension. Furthermore, often the eardrum is reconstructed in the context of ossiculoplasty or the prosthesis head plate is covered with cartilage. This procedure modifies the elastic properties and the stiffness of the lateral components in comparison to the natural tympanic membrane with the manubrium. Own investigations, however, could show that cartilage with a thickness of 500 µm in the area of the deformation as it is caused by a clinically relevant prolongation of the prosthesis ranges in the linear visco-elasticity and thus an expansion without measurable increase of the stiffness takes place.

In summary, for ear surgeons this means that from a biomechanical point of view any pretension should be avoided in order not to cause transmission losses already at the beginning that could be enhanced by further, biological factors. Undoubtedly, the surgical reality requires positioning and coupling of prostheses as stable as possible, so that under certain circumstances in the operation room the generation of pretension cannot be completely avoided. Nonetheless, the surgeon should know about those correlations and apply methods of dislocation protection for stabilization of the prostheses in favor of a reduction of the pretension [97], [106], [107], [122].

## Notes

### Competing interests

The authors declare that they were supported by Heinz Kurz Medical Technique Company during the last 3 years in the context of travelling grants and performed contract research.

## Figures and Tables

**Table 1:**
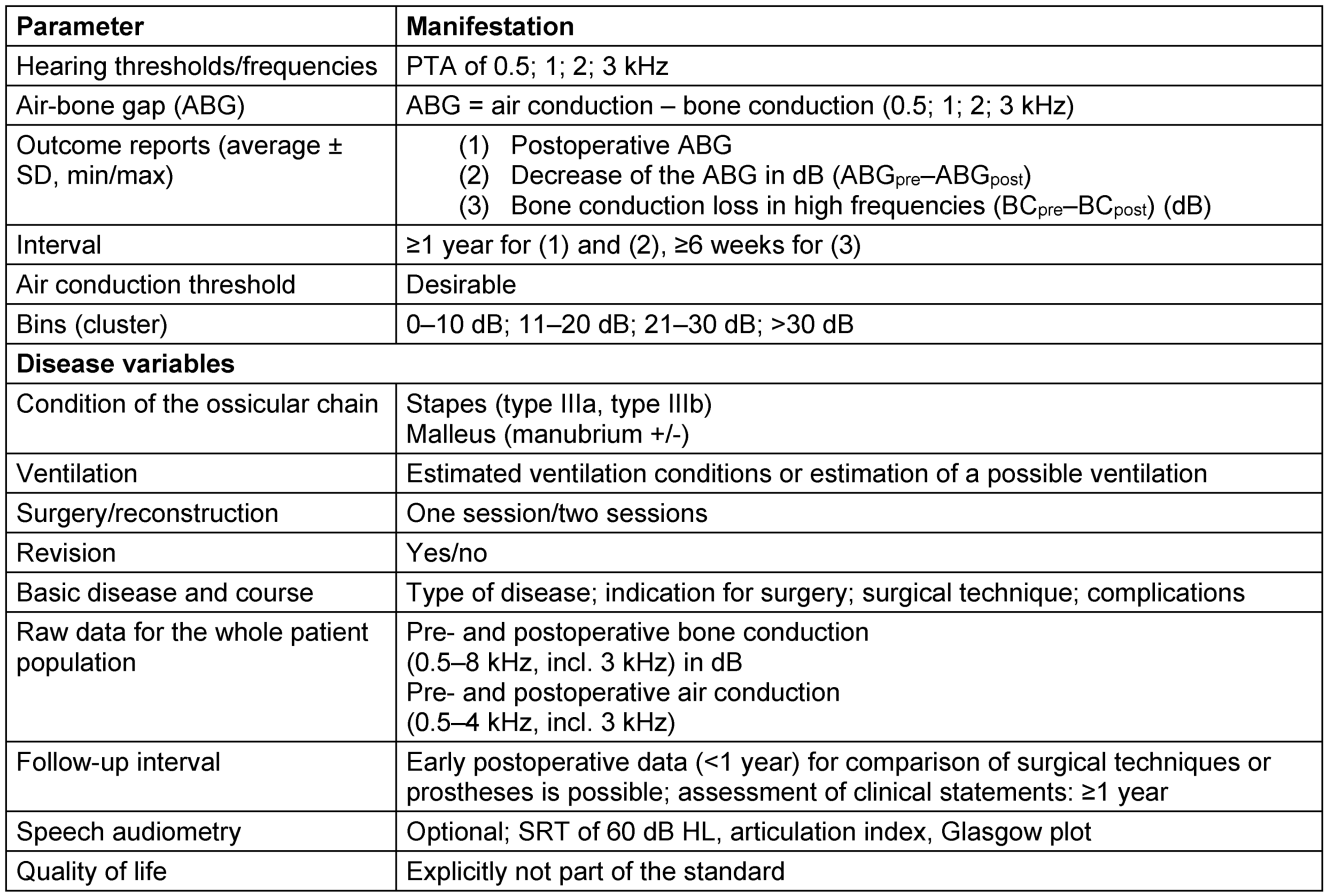
Recommendations of the AAO-HNS for documentation of hearing results in studies [13] (1995).

**Table 2:**
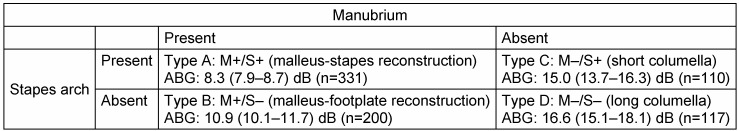
Classification of the status of the defective ossicles according to Austin [39]. The fourfold table displays the combinations of the 4 possible conditions of manubrium (M) and stapes arch (S), i.e. type A–D, as well as the recommended reconstruction technique. To better describe the findings, the PTA-ABG values (average value and 95% CI interval) stratified according to the Austin types, are given by Stankovic [63]. The clearly lower ABG values of the Austin types A and B indicate the significance of the manubrium for the hearing outcome.

**Table 3:**
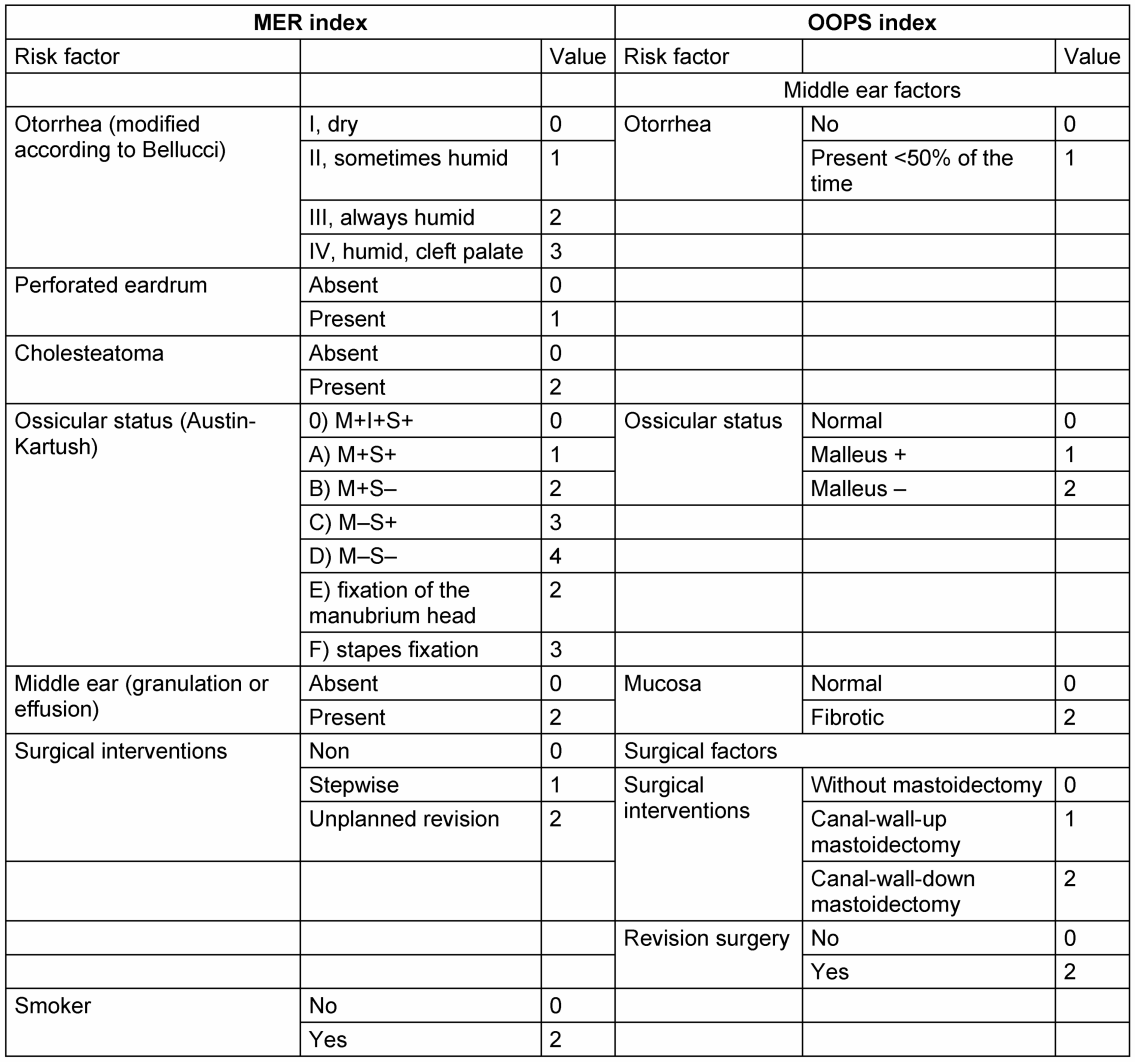
This table displays the middle ear risk (MER) index and the ossiculoplasty outcome parameter staging (OOPS) index. For each risk factor, corresponding scores were allotted that were summed up. For the MER index, the categories from 0–3 (light), 4–6 (moderate), and 7–12 (severe) disease were applied. The higher the total score was in the according index, the more difficult is it to determine the disease and the poorer is the prognosis for the reduction of the postoperative air-bone gap (M: malleus; I: incus:, S: stapes; +: present; –: missing).
